# Navigating a complex landscape – A review of the relationship between inflammation and childhood trauma and the potential roles in the expression of symptoms of depression

**DOI:** 10.1016/j.bbih.2022.100418

**Published:** 2022-01-19

**Authors:** Courtney Worrell

**Affiliations:** Stress, Psychiatry and Immunology Laboratory, Department of Psychological Medicine, Institute of Psychiatry, Psychology & Neuroscience, King's College London, UK

**Keywords:** Major depressive disorder (MDD), Childhood trauma, Inflammation

## Abstract

Recent research has explored associations between depression and inflammation, and interactions between childhood trauma and both factors. Major Depressive Disorder (MDD) is a prevalent issue and with one third of patients not responding to standard antidepressant treatments, it is crucial to develop our understanding. While research delves into the complex landscape of the roles of both childhood trauma and inflammation in depression, it is customary for literature to explore effects on presence of depression. However, understanding if childhood trauma and inflammation may be affecting the symptom profiles of depression and what implications this may have, is lacking.

## 1 Introduction

Childhood trauma can be conceptualised as any experience of low socioeconomic status, bereavement, bullying, facing emotional or physical neglect, physical or sexual abuse and far more, in childhood. Affects are carried through life, often increasing risk of negative health outcomes in the long-term and therefore a public health problem (Schnyder, 2013; World Health Organisation; Magruder et al., 2017). It has always been a challenge to quantify prevalence, however it's suggested that one in five adults may have experienced childhood trauma (Crime Survey for Child ab, 2020). Following the COVID-19 pandemic there are fears that numbers may have increased, with restrictions across the globe potentially increasing risk of experiencing abuse or neglect, and reducing opportunity to seek refuge at school. However, translation to scientific data is not yet clear, thus this should be considered speculation. More time at home may have also been beneficial for other children and reduced schoolground bullying, for example. Nonetheless, we should be aware of the potential of a new wave of children affected, possibly creating a group with substantially increased risk of negative outcomes as they continue through life.

Shonkoff and Garner (2012) describe an ‘ecobiodevelopmental framework’ arguing ‘many adult diseases should be viewed as developmental disorders that begin early’ due to the influence early life stress, thus demonstrating the enormity of the consequences (Shonkoff and Garner, 2012). Numerous studies have identified associations between trauma in early years and development of later pathology, with Kessler et al. (2010) reporting it may account for 29.8% of all psychopathology disorders, inclusive of mood, anxiety, behavioural and substance disorders (Kessler et al., 2010). The reason for such continual impact is likely due to dramatic imprint at a time of vulnerability for the developing brain and immune systems. This impact is demonstrated clearly through animal models (Danese and Lewis, 2016). One pattern observed with childhood trauma is elevated levels of inflammation – reportedly due to the impact of psychological stress triggering inflammatory responses during this critical period of development (Danese and Lewis, 2016). Danese's earlier work (Danese et al., 2007) was some of the first to identify cumulative exposure, with more trauma experienced associated with higher-graded inflammation after 20 years, uninfluenced by adult stress, socioeconomic exposure, and health. However, a recent systematic review found that childhood maltreatment is most consistently associated with C-reactive protein (CRP) as an inflammatory biomarker, but other biomarkers are reported less consistently, potentially due to methodological disparities (Kerr et al., 2021). In studies finding associations, patterns of trauma and inflammation were found to be strongest for those who developed depression later in life (Danese et al., 2008). Notably, associations with childhood trauma and inflammation have been identified independently to depression and other clinical comorbidities (Coelho et al., 2013).

Major depressive disorder (MDD) is a complex condition affecting millions of individuals worldwide with costs extending the individual to additionally public health, increasing both morbidity and mortality (Kessler and Bromet, 2013). Diagnostic and Statistical Manual of Mental Disorders -5 (DSM-5) outlines that MDD can be categorised by at least a 2 week period of symptoms such as depressed mood, anhedonia, fatigue or loss of energy, appetite disturbance or weight change, diminished ability to concentrate and more (American Psychiatric Association, 2013). It has been persistently found that childhood trauma is linked with increased risk for developing depression in later life, with some findings suggesting that there was also a variation in the severity of depression dependent on the type of trauma reported (Humphreys et al., 2020).

Rich bodies of literature have reported on the association between inflammation and pathology, with elevated levels of inflammation identified in a range of psychiatric disorders including, but not restricted to, anxiety, post-traumatic stress disorder, psychosis and more (Baumeister et al., 2014; Naudé et al., 2018; Michopoulos et al., 2017; Gill et al., 2009; Di Nicola et al., 2013). One of the most consistent findings is the link between inflammation and depression. Data consistently demonstrates that a subset of patients with depression show elevated levels of inflammatory biomarkers including Interleukin-6 (IL-6), Interleukin-1Beta (IL-1β), Tumour Necrosis Factor-alpha (TNF- α) and CRP (Miller and Raison, 2016). Of note is that these findings are only observed in a subset of patients (Chamberlain et al., 2019) and there are several theories for the differentiation, elevated inflammation for treatment-resistant depressed patients being one. In this paper by Chamberlain et al. (2019), treatment-resistance was defined as participants with depressive symptoms, as measured by the Hamilton Rating Scale for Depression, HAM-D_17,_ and who were receiving monoaminergic antidepressant treatment at adequate dose for at least 6 weeks. While this study places treatment resistance in the context of pharmacological treatments, it is important to note that depression can occur in a spectrum of severities and many treatments, including non-pharmacological, are available and beneficial, although beyond the scope of the current review.

Our research group were recently able to demonstrate that treatment-resistant patients had increased inflammasome activation (Cattaneo et al., 2020). Treatment-resistant and drug-free participants had higher pro-inflammatory cytokines and chemokines, and purinergic receptor P2RX7 expression, and lower glucocorticoid resistance, compared to responders. This is particularly significant as approximately one third of depressed patients do not find improvement from pharmacological treatments (Strawbridge et al., 2015). The association is clear however, the exact biological mechanisms and directionality of the relationship lacks clarity. With evidence demonstrating that childhood trauma increases risk of depression, and individual associations with inflammation, our understanding of how the three factors come together is clouded.

What remains unclear is the exact relationship between inflammation, depression, and childhood trauma, and furthermore, much literature explores associations with presence or absence of MDD as opposed to the specific symptom profiles. This is an area of great interest to me. This is a short qualitative review aiming to reflect on emerging research navigating the complex landscape of depression and inflammation, and the possible role of childhood trauma with a specific view to symptomology, and additionally highlighting key findings from my research group.

## 2 Childhood trauma, inflammation, and presence of depression

When it comes to research into maltreatment, we see different optimisation dependent on aims, for example we may look at the severity of trauma as scales, or a binary outlook – presence versus absence. Similarly, a significant proportion of the research explores presence or absence of MDD.

Several studies observe associations between childhood trauma and inflammation in MDD participants. Grosse et al. (2016) explored stress mechanisms behind inflammation and depression, comparing effects of childhood trauma and recent stress in a sample of participants with MDD, compared with healthy controls in order to investigate whether increased inflammation specifically occurs in patients with a history of stress. A total of 327 inpatients with a current diagnosis of MDD were recruited which may suggest increased severity of depression and could affect results (Grosse et al., 2016). The participants were each treated according to their psychiatrists’ usual recommendation. Levels of IL-6 and TNF-α, as measured in serum, demonstrated that overall inflammatory levels were not associated with childhood trauma or stress from later life experiences (Grosse et al., 2016). However, interestingly higher severity of sexual abuse in childhood (measured by the Childhood Trauma Questionnaire, or CTQ) was associated with higher levels of IL-6 and TNF-α in patients with MDD, leading to conclusions of a linear relationship with childhood sexual abuse and increased inflammatory cytokines in MDD patients. There were no associations with emotional or physical abuse, emotional or physical neglect (Grosse et al., 2016). Increased IL-6 and TNF-α are consistently reported in adults with a history of childhood trauma, and type of trauma has been associated with different outcomes in a meta-analysis performed by Baumeister et al. (2016) highlighting evidence that individual types of trauma have differential impact on inflammatory markers (Baumeister et al., 2016). It could be speculated that seemingly consistent associations with sexual abuse and IL-6 and TNF-α specifically, may be a result of longer lasting effects of severe forms of childhood trauma. The authors of the meta-analysis suggest that beyond childhood sexual abuse alone, more physical forms of abuse (sexual abuse and physical abuse) are found to be significantly associated with IL-6 and TNF-α. Our group recently performed an updated systematic review using the same search criteria as Baumeister et al. and when looking specifically at IL-6, reported that total scores for childhood trauma on the CTQ provided varying results, however a number of studies did identify a significant association between abuse (including sexual, physical and emotional abuse) and increased levels of IL-6, however no such association was identified for neglect (physical and emotional neglect) (Brown et al., 2021). Grosse et al. importantly discuss that many participants who report childhood sexual abuse also report other forms of abuse (Grosse et al., 2016), and thus we should consider the possible implications of this too. The methodological features of the reviewed literature can be seen in Table 1.

**Table 1:** Methodological features of reviewed literature.

Author	Sample size	Inflammatory biomarkers/biological measure	Childhood trauma measure	Depression measure	Main findings
Grosse et al., 2016	394	TNF-α, IL-6	CTQ	MINI & IDS	Inflammatory levels not associated with CT or later life stress but higher CT scores for sexual abuse associated with increase IL-6 & TNF- α
Lu et al., 2013	65	120 array cytokine antibody – reported 13: AgRP, b-FGF, BTC, GITR-L, I-TAC/CXCL11, IL-1β, IL-1 R1, MEC/CCL28, NT-4, TGF-β3, TECK/CCL25, TRAIL-R4, VEGF	CTQ	SCID	MDD associated with higher levels of 13 cytokines but attenuated when CT factored
Schiweck et al., 2020	414	IL-6, hsCRP Monocyte gene expression of ADM, ATF3, BCL2A1, BTG3, CCL2, CCL20, CCL7, CD9, CDC42, CXCL2, DHRS3, DUSP2, EMP1, EREG, FABP5, HSPA1A/HSPA1B, IL-1α, IL-1β, IL1R1, IL-6, IRAK2, MAFF, MAPK6, MXD1, NAB2, PDE4B, PTGS2, PTPN7, PTX3, SERPINB2, STX1A, THBD, TNF, TNFAIP3	CTQ short form	MINI, IDS-C & IDS-SR	Higher prevalence of CT in MDD groups associated with higher inflammatory gene expression. No difference between CT vs no CT
Danese et al., 2008	1000	hsCRP	Parental reports, prospective behavioural observations & retrospective reports	DIS	MDD & CM associated with increased levels of hsCRP vs controls
Negele et al., 2015	349	N/A	CTQ German short version	SCID I, SCID II & QIDS-C	Multiple CT experiences associated with more severe depressive symptoms
Hughes et al., 2013	78	TLR4, TREM-1, IL-1 β, COX-2, IL-4	CTQ	HAM-D	Depression severity associated with increased TLR4 & TREM-1, & CT scores associated with IL-1 β, COX-2 & TLR4
Nikkheslat et al., 2020	218	hsCRP Cortisol (HPA-axis activity)	CTQ	HAM-D & SCID	Severity of CT associated with treatment resistance & increased HPA-axis activity & cortisol production (especially in depression with glucocorticoid resistance)
Chamberlain et al., 2019	252	hsCRP	CTQ	HAM-D & SCID	Increased hsCRP associated with clinical phenotypes of depression including vegetative depressive symptoms, BMI, state anxiety ​& CT items
Van Veen et al., 2013	2615	N/A	Semi-structured childhood trauma interview	CIDI & MASQ-D30	Emotional neglect associated with 3 symptom dimensions; physical & sexual abuse associated with general distress & anxious arousal; physical abuse associated with anxious arousal
Moskvina et al., 2007	354	N/A	CTQ	SCAN	Earlier age of onset for depression associated with more CT. CT & depression (or siblings of depressed) were more likely to have cognitive symptoms and psychomotor retardation
Matza et al., 2003	4907	N/A	5 items from NCS	CIDI 1.0	CT associated with atypical depression
Lamers et al., 2013	776	CRP, IL-6, TNF-α Cortisol (HPA-axis activity)	N/A	CIDI 2.1	Atypical depression associated with higher levels of CRP, IL-6 & TNF- α vs melancholic depression and controls
Yoon et al., 2012	105	IL-2, IL-4, IL-6, TNF-α	N/A	SCID	Atypical depression associated with lower levels of IL-4 & IL-2. IL-6 & TNF-α showed no significant difference vs controls
Glaus et al., 2014	3719	CRP	N/A	DIGS	Atypical depression associated with higher levels of hsCRP. Association remained for men after adjustment, but not females
(Mohamed et al., 2020)	98		Not applicable	SCID & BSS	Atypical depression associated with increased CRP & increased suicidality

Abbreviations – ADM ​= ​Adrenomedullin, AgRP = Agouti-related protein, ATF3 ​= ​Activating transcription factor 3, BCL2A1 ​= ​BCL2 Related Protein A1 b-FGF ​= ​basic Fibroblast Growth Factor, BMI = Body Mass Index, BSS = Beck Scale for Suicidal Ideation, BTC = Betacellulin, BTG3 ​= ​B-cell-translocation gene 3, CA = Childhood adversity, CCL2 ​= ​C-C Motif Chemokine Ligand 2, CCL20 ​= ​C-C Motif Chemokine Ligand 20, CCL7 ​= ​C-C Motif Chemokine Ligand 7, CD9 ​= ​CD9 Molecule, CDC42 ​= ​Cell division control protein 42 homolog, CIDI = The Composite International Diagnostic Interveiw, CM = Childhood maltreatment, COX-2 ​= ​Cyclooxygenase-2, CRP = C-Reactive Protein, CT = Childhood trauma, CTQ = Childhood Trauma Questionnaire, CXCL2 ​= ​C-X-C Motif Chemokine Ligand 2, DHRS3 ​= ​Dehydrogenase/Reductase 3, DIGS ​= ​Diagnostic Interview for Genetic Studies, DIS ​= ​Diagnostic Interview Schedule, DUSP2 ​= ​Dual Specificity Phosphatase 2, EMP1 ​= ​Epithelial membrane protein 1, EREG ​= ​Epiregulin, FABP5 ​= ​Fatty Acid Binding Protein 5, GITR-L ​= ​Glucocorticoid Induced Tumour Necrosis Factor-ligand, HAM-D ​= ​Hamilton Rating Scale for Depression, HPA-axis ​= ​Hypothalamic Pituitary Adrenal axis, hsCRP ​= ​High sensitivity C-Reactive Protein, HSPA1A/HSPA1B ​= ​Heat Shock Protein Family A Member 1A/Member 1B, IDS = Inventory of Depressive Symptomatology, IDS-C = Inventory of Depressive Symptomatology - Clinician-Rated, IDS-SR = Inventory of Depressive Symptomatology - Self-Reported, IL-1α ​= ​Interleukin-1 alpha, IL-1β ​= ​Interleukin-1 Beta, IL1-R1 ​= ​Interleukin1 – Receptor 1, IL-4 ​= ​Interleukin-4, IL-6 ​= ​Interleukin-6, IRAK2 ​= ​Interleukin 1 Receptor Associated Kinase 2, I-TAC/CXCL11 ​= ​Interferon induced T cell α chemoattractant/CXC Motif Chemokine Ligand 11, MAFF ​= ​MAF bZIP transcription factor F, MAPK6 ​= ​Mitogen-Activated Protein Kinase 6, MASQ-D30 ​= ​Mood and Anxiety Symptoms Questionnaire-Dutch adaptation 30-item, MXD1 ​= ​MAX Dimerization Protein 1, MEC/CCL28 ​= ​Mucosae-associated epithelial chemokine/C-C Motif Chemokine Ligand 28, MINI ​= ​Mini-International Neuropsychiatric Interview, NAB2 ​= ​NGFI-A Binding Protein 2, NCS = US National Comorbidity Survey, NT-4 ​= ​Neurotrophin-4, PDE4B ​= ​Phosphodiesterase 4B, PTGS2 ​= ​Prostaglandin-Endoperoxide Synthase 2, PTPN7 ​= ​Protein Tyrosine Phosphatase Non-Receptor Type 7, PTX3 ​= ​Pentraxin 3, QIDS-C ​= ​Quick Inventory of Depression Symptomatology – Clinical-Rated, SCAN = Schedules for Clinical Assessment in Neuropsychiatry, SCID = Structured Clinical Interview for DSM, SERPINB2 ​= ​Serpin family B Member 2, STX1A ​= ​Syntaxin 1A, TECK/CCL25 ​= ​Thymus-expressed chemokine/C-C Motif Chemokine Ligand 25, TGF- β3 ​= ​Transforming growth factor - β 3, THBD ​= ​Thrombomodulin, TLR4 ​= ​Toll-like receptor 4, TRAIL-R4 ​= ​Tumour Necrosis Factor related apoptosis inducing ligand-receptor 4, TNF ​= ​Tumour Necrosis Factor, TNF- α ​= ​Tumour Necrosis Factor-alpha, TNFAIP3 ​= ​TNF Alpha Induced Protein 3, TREM-1 ​= ​Triggering receptor expressed on myeloid cells 1, VEGF = Vascular endothelial growth factor.

Other studies have explored a wider cytokine array, including Lu et al. (2013) who tested the assumption that several cytokines may be associated with childhood trauma in MDD; childhood trauma was confirmed using the CTQ and depression confirmed using the Structured Clinical Interview for DSM-IV (SCID). Measured in plasma, the authors used a novel cytokine antibody array and found individuals with MDD had higher levels of 13 cytokines and growth factors including agouti-related protein (AgRP), basic fibroblast growth factor (b-FGF), betacellulin (BTC), glucocorticoid-induced tumour necrosis factor-ligand (GITR-L), interferon induced T-cell α chemoattractant (I-TAC/CXCL11), interleukin-1β (IL-1β), interleukin-1 Receptor 1 (IL-1 R1), mucosae-associated epithelial chemokine (MEC/CCL28), neurotrophin-4 (NT-4), transforming growth factor-β3 (TGF-β3), thymus-expressed chemokine (TECK/CCL25), tumour necrosis factor related apoptosis inducing ligand-receptor 4 (TRAIL-R4), and vascular endothelial growth factor (VEGF) in comparison with controls when looking retrospectively at the associations (Lu et al., 2013). However, when childhood trauma was factored into the model, these significant differences were lost. Notably, this was performed in a relatively small sample of 65 participants. Furthermore, case-control studies exploring inflammatory gene expression demonstrate that childhood adversity was associated with raised inflammatory gene expression in MDD patients but not in healthy controls (Schiweck et al., 2020). In this study, MDD was confirmed using the Mini International Neuropsychiatric Interview (MINI).

Work of Danese et al. (2008) demonstrated in a prospective longitudinal cohort that participants with current MDD, as measured by the Diagnostic Interview Schedule (DIS), and history of childhood maltreatment were more likely to have increased levels of hsCRP than nondepressed participants, suggesting that in this sample, childhood trauma contributed to the co-occurrence of depression and inflammation (Danese et al., 2008). This finding was again based on a diagnosis of current MDD as opposed to symptom severity.

While understanding that childhood trauma may appear to have some role in the co-occurrence of MDD and inflammation, it would also be significant to improve understanding of how these factors may affect MDD itself. We can first look at this with regards to the severity of the symptoms reported.

## 3 Childhood trauma, inflammation and symptom severity in depression

Severity of depression is important to explore considering childhood trauma impacts outcome. When looking at the severity of depressive symptoms in a sample with MDD or dysthymia according to DSM-IV, research showed that experiences of multiple types of childhood trauma was associated with higher severity symptoms (Negele et al., 2015). Further, type of trauma impacted the severity of symptoms, with emotional and sexual abuse associated with higher severity (Negele et al., 2015). However, what is interesting to explore is how inflammation comes into this clinical picture.

In a small cross-sectional study comparing patients with depression and healthy controls, positive correlations were identified between depression severity (as measured on the clinician-administered HAM-D_17_) and inflammatory markers Toll-Like Receptor 4 (TLR4) and Triggering Receptor Expressed on Myeloid Cells 1 (TREM-1), depression severity and total CTQ scores, and total CTQ scores and IL-1 β, Cyclooxygenase-2 (Cox-2), and TLR4. Authors concluded childhood adversity is associated with a dysregulated immune profile forming risk for depression (Hughes et al., 2013).

Interestingly, childhood trauma has been associated with sub-optimal response to pharmacological antidepressant treatments, which ties in with theories of treatment resistance. Work from our group, the Stress, Psychiatry and Immunology (SPI) lab, demonstrated that treatment-resistant participants were more likely to have been exposed to childhood trauma than those who responded to antidepressants (Nikkheslat et al., 2020). In this study, ‘non-responders’ included treatment with 1+ monoaminergic antidepressants for at least 6 weeks and who had a HAM-D_17_ score over 13, compared with ‘responders’ scoring less than 7. Non-responders had higher CTQ scores. Addressing childhood trauma as a risk factor for depression and association with poor treatment response to antidepressant treatments, the study additionally explored Hypothalamic-pituitary-adrenal axis (HPA-axis) function, linked to both childhood trauma and depression, as a main focus. The authors reported previous observations that glucocorticoid resistance is observed in MDD patients with coexistence of inflammation. Results showed that in those with decreased glucocorticoid functioning, severity of childhood trauma was associated with increased diurnal cortisol levels, leading to conclusions that childhood trauma contributes to increased HPA-axis hyperactivity in patients with glucocorticoid resistance. While main findings focus on treatment response, this categorisation provides information surrounding symptom severity. In the same sample, Chamberlain et al. (2019) explored treatment-resistant depression and peripheral hsCRP (Chamberlain et al., 2019). CRP was significantly elevated in the treatment-resistant group compared to healthy controls. Further analysis explored clinical phenotypes, identifying certain items on the CTQ, feeling unloved during childhood and wanting to change their family, were associated with higher CRP (Chamberlain et al., 2019).

We already see a gap in the literature, with more focus placed on a presence or absence of depression than symptom severity. There is a further gap when it comes to the specific symptoms expressed.

## 4 Core gaps in our knowledge - Childhood trauma, inflammation and depressive symptoms

Research lacks regarding clinical presentations, and relatively few studies explore effect on the type of symptoms expressed in this complex association.

MDD is complex with numerous phenotypes presenting a clinical reality, and subtyping MDD profiles can be useful. A meta-analysis by Harald and Gordon (2012) describes subtyping as an attempt to overcome non-specificity of MDD diagnoses and has potential implications for treatment, although further research is needed (Harald and Gordon, 2012). It is becoming more common for research to embrace this heterogeneity when investigating the disorder.

Literature suggests that childhood trauma, and more specifically different types of trauma, are associated with different symptom dimensions of depression. A cross-sectional study using the tripartite model of depression and anxiety (Clark and Watson, 1991) found that three of the four types of trauma (emotional neglect, psychological and sexual abuse) were associated with increased levels of general distress in a longitudinal cohort (Van Veen et al., 2013). Interestingly, emotional neglect was strongly associated with increased anhedonic depression, defined as an absence of positive mood states, compared to healthy controls (Van Veen et al., 2013). Other studies have looked specifically at symptom dimensions of depression which were identified using the DSM-IV and/or International Classification of Diseases-10 (ICD-10), as opposed to the tripartite model, finding participants with childhood trauma (experienced two or more episodes of unipolar depression, or were siblings of depressed patients) were more likely to experience symptoms of cognitive and psychomotor retardation than those with no trauma (Moskvina et al., 2007). While based on association, authors to speculate on cause-and-effect. There was no significant difference in dimensions dependent on the type of trauma experienced, however it was reported emotional abuse was associated with these depressive symptoms (Moskvina et al., 2007). The authors additionally note no associations were identified with atypical symptomology as reported in the literature.

Atypical depression, defined as a diagnosis of Major Depressive Episode (MDE) in addition to atypical symptoms based on DSM-IV, refers to the expression of symptoms including mood reactivity, appetite changes, sleep characteristics and feeling weighed down, and has been reported to be associated with childhood trauma compared to non-atypical depression (including melancholic depression) and healthy controls (Matza et al., 2003). This finding was most significant for emotional neglect and sexual abuse. Notably, this study did not look at childhood trauma alone and the atypical group were additionally more likely to experience psychiatric comorbidities, have a history of parental depression and experience disability.

There has been much research exploring the relationship between atypical depression and inflammation. A study using data from a longitudinal naturalistic cohort study focussed on atypical versus melancholic depression as two differential subtypes of MDD which were classified by the clinician-administered Composite International Diagnostic Interview (CIDI), and showed atypical depression was associated with increased CRP, IL-6 and TNF-α in comparison to melancholic depression and healthy controls (Lamers et al., 2013). The key distinction between subtypes being that atypical is akin to over-eating and weight gain, versus loss of appetite and weight loss in melancholic. However, increased levels of IL-6 and TNF-α are not always reported. Yoon et al. (2012) explored multiple cytokines in atypical depression and showed no difference in IL-6 and TNF-α compared to melancholic depression. Notably this was a relatively small sample (n ​= ​105) of participants diagnosed with the SCID (Yoon et al., 2012). Findings between inflammation and atypical depression are not entirely consistent, with one study demonstrating atypical MDD was associated with increased hsCRP compared to melancholic combined atypical-melancholic and unspecified subtypes until the models controlled for covariates (Glaus et al., 2014). The sample included general population in a cohort study agreeing to psychiatric evaluation.

Further studies have found elevated levels of CRP in participants with atypical depression when compared to anxious, melancholic, psychotic, and unspecified depression, diagnosed using the SCID for DSM-V (Mohamed et al., 2020). Additionally, this group had a significant increase in suicidality. This is particularly interesting as the atypical depression group in Matza et al. (2003) exploring childhood trauma, was additionally associated with increased anhedonia and greater suicidality compared to the non-atypical group and healthy controls (Matza et al., 2003).

As discussed, there is a lot of research into the interaction between these three factors, that remain, for the most part, separate. Research exploring how the three come together thus far largely looks at associations within the presence and absence of MDD.

As we have seen with research into inflammation and treatment-resistant depression there are potential implications for course of illness, along with the additional effect of childhood trauma on risk and outcomes. Bettering our understanding of the biological mechanisms could open possible routes for novel treatments. Additionally, furthering understanding of whether childhood trauma and inflammatory profiles may influence the expression of depressive symptoms arms us with an opportunity for best supporting these individuals. Research so far suggests that inflammation affects symptom expression in depression and so does childhood trauma, so a next step would be to bring these factors together in the overall picture. The nature of this very sensitive and distressing scenario is important here, and we have seen implications for suicidality; particularly prevalent as childhood sexual abuse for example, may be associated with increased suicidality in depression as early as in adolescence (Stewart et al., 2015; Miller et al., 2013).

## 5 Limitations

While this review begins to reflect on findings exploring the complicated interaction of childhood trauma, inflammation and depression, it is important to consider that this is a short reflection which did not use systematic methods. It should be noted in this regard, that the scope of the review did not encompass different depressive diagnoses, nor comorbidities which warrant further investigation. Furthermore, it is important to highlight that there are complexities in this topic not addressed in the scope, including but not limited to: different experiences of childhood trauma such as age, duration, reporting and more, treatment differences in depression and responses. Thus, further in-depth reviews of this important topic are needed in order to advance our understanding.

## 6 Conclusion

While the presence of inflammation has established links with both depression and childhood trauma, exact mechanisms are not fully understood. Research is needed to unpick the ties between childhood trauma, depressive symptoms, and inflammation – is childhood trauma a contributing factor to increased inflammation in a subgroup of depressed patients, and are such subgroups demonstrating differential symptom profiles? Lines of research need to converge, determining how factors interact, with a view to identifying new intervention approaches for this clinical scenario, or providing appropriate support to those exposed to childhood trauma, knowing the risk carried with them into later life.
